# Addition of antimicrobials to oral sprays containing nonsteroidal antiinflammatory drugs does not reduce the severity of postoperative sore throat: a prospective, randomized, placebo-controlled trial

**DOI:** 10.55730/1300-0144.6044

**Published:** 2025-07-08

**Authors:** Pelin YILDIZ, Mobin HEMMATI, Leyla KUTLUCAN, Tankut UZUN, Togay MÜDERRİS

**Affiliations:** 1 Department of Otorhinolaryngology, Faculty of Medicine, İzmir Bakırçay University, Çiğli Training and Research Hospital, İzmir, Turkiye; 2 Department of Anesthesiology and Reanimation, Faculty of Medicine, İzmir Bakırçay University, Çiğli Training and Research Hospital, İzmir, Turkiye

**Keywords:** Sore throat, oral sprays, nonsteroidal antiinflammatory agents, antiinfective agents, randomized controlled trial

## Abstract

**Background/aim:**

Postoperative sore throat (POST) is a common complication following general anesthesia that significantly affects patient satisfaction, prolongs recovery, and increases treatment costs. This study aimed to evaluate whether the addition of antimicrobial agents to NSAID-based oral sprays could enhance the preventive efficacy against POST.

**Materials and methods:**

In this prospective, randomized, placebo-controlled trial, 105 patients (ASA I–II; age 18–65 years) scheduled for elective ear surgery under general orotracheal anesthesia were enrolled. Patients were randomly allocated into three groups: a placebo group; a flurbiprofen oral spray group; and a group receiving an oral spray containing benzydamine hydrochloride, chlorhexidine digluconate, and cetylpyridinium chloride. The sprays were administered under direct laryngoscopy before intubation and after the final oropharyngeal aspiration. POST severity was assessed using a 10mm Visual Analog Scale (VAS) at 1 h, 6 h, 24 h, and 1 week postoperatively. Patients were also subgrouped based on surgical duration (<120 min vs. ≥120 min).

**Results:**

Both NSAIDbased treatments significantly reduced VAS scores at early postoperative time points compared to the placebo. In subgroup analysis, patients undergoing surgeries lasting less than 120 min exhibited lower VAS scores with both active treatments, while in those with surgeries ≥120 min, significant differences were noted at 1 and 6 h. No significant difference was found between the flurbiprofen spray and the combination spray.

**Conclusion:**

NSAIDcontaining oral sprays effectively reduce the severity of postoperative sore throat. However, the addition of antimicrobial agents does not provide extra analgesic benefit, suggesting that simpler, costeffective NSAID formulations may be preferable in clinical practice.

## 1. Introduction

Postoperative sore throat (POST) is one of the most commonly reported complications following general anesthesia, with an incidence of up to 62% [1]. POST negatively impacts patient satisfaction, prolongs recovery, and increases treatment costs [2]. It is a broadly defined term encompassing various conditions, including pharyngitis, laryngitis, tracheitis, coughing, hoarseness, and dysphagia, which manifest in the early postoperative period [2,3]. Several risk factors for POST development have been identified, including female sex, younger age, preexisting pulmonary disease, prolonged anesthesia, tracheal tube size, the use of double-lumen endotracheal tubes (ETT), presence of blood-stained ETT at extubation, and excessive cuff pressures over 20 cmH_2_O [4]. The etiology of POST is complex, involving mucosal injury, inflammation, airway trauma, ischemia, and gastric content regurgitation, among other factors [5]. Several pharmacological interventions have been proposed to prevent or alleviate POST, including corticosteroids, topical sprays, nonsteroidal antiinflammatory drugs (NSAIDs), and N-methyl-D-aspartate (NMDA) receptor antagonists [6].

Benzydamine is a nonsteroidal antiinflammatory drug (NSAID) with analgesic and local anesthetic properties. It reduces inflammation and pain by inhibiting the production of proinflammatory chemicals, such as prostaglandins, and treating pain and inflammation in the mouth, throat, and gums (e.g., sore throat, tonsillitis, oral ulcers) [7,8]. It is commonly used as a topical treatment in mouthwashes, sprays, and gels to promote oral health and has been found to be effective in reducing the severity of POST [1,2,3,9]. Chlorhexidine is a broad-spectrum antimicrobial agent used to reduce bacteria and other microorganisms. It disrupts microbial cell membranes, leading to the destruction of bacteria and some fungi and viruses [10,11,12]. It is used in mouthwashes to treat gingivitis and periodontitis and to prevent oral infections, used as a disinfectant for wounds or as a surgical hand scrub, and applied to clean catheters or surgical equipment to prevent infections [13]. Cetylpyridinium chloride is a quaternary ammonium compound with antiseptic and antimicrobial properties [14]. It disrupts the bacterial cell membrane, causing leakage of cell contents and death of the bacteria [15]. It is used in cleaning solutions for its antibacterial properties, often included in sprays and lozenges to soothe sore throats and reduce microbial load [16,17]. Flurbiprofen is a well-established NSAID with analgesic properties, and has been demonstrated to effectively reduce sore throat both in lozenge and oral spray formulations, and is effective in reducing POST [18].

The ETT is recognized as an independent risk factor for infection in intubated patients, with studies demonstrating that these tubes become colonized within 24 h [19,20]. Based on the hypothesis that bacterial colonization may exacerbate inflammation, thereby increasing the severity and incidence of postoperative sore throat, we aimed to evaluate whether adding antimicrobials to antiinflammatory sprays could enhance their preventive efficacy. Therefore, this study compared the effectiveness of oral sprays containing benzydamine hydrochloride, chlorhexidine digluconate, and cetylpyridinium chloride with those containing flurbiprofen or a placebo in preventing postoperative sore throat.

## 2. Materials and methods

This prospective study was conducted at the Otorhinolaryngology Department of İzmir Bakırçay University Hospital, between January 2024 and October 2024. A total of 105 patients with ASA physical status I or II, aged 18 to 65, undergoing elective ear surgery under general orotracheal anesthesia were enrolled. Patients who experienced vomiting during the study period, required additional postoperative analgesics, presented with preoperative sore throat and hoarseness, or underwent one or more unsuccessful intubation attempts were excluded.

Patients were randomly assigned to three groups of 35 each: (1) placebo (P); (2) flurbiprofen oral spray (F); and (3) benzydamine HCl–chlorhexidine digluconate–cetylpyridinium chloride oral spray (B).

Randomization was performed using a computer-generated randomization code with block randomization. A copy of the codes was archived at the Department of Biostatistics, and another copy was provided to the nurse responsible for preparing the sprays. The flurbiprofen, benzydamine HCl–chlorhexidine digluconate–cetylpyridinium chloride, and placebo sprays were prepared in the otolaryngology clinic by an experienced nurse, packed into identical 30-mL bottles, and labeled as A, B, or C according to the randomization scheme.

The commercial formulations used were as follows:

✓ **Flurbiprofen oral spray:** A 30-mL bottle contained 0.075 g of flurbiprofen, with each spray delivering 0.13 mL (0.325 mg).
✓ **Benzydamine hydrochloride, chlorhexidine digluconate, and cetylpyridinium chloride oral spray:** A 30-mL bottle contained 0.045 g of benzydamine hydrochloride (each spray: 0.18 mL, 0.270 mg), 0.036 g of chlorhexidine digluconate (each spray: 0.216 mg), and 0.015 g of cetylpyridinium chloride (each spray: 0.09 mg).
✓ **Placebo solution:** Prepared from distilled water and transferred into identical 30-mL bottles

All sprays were delivered to the operating room before anesthesia induction according to the randomization scheme. Administration of the sprays to the oropharynx and hypopharynx was performed under direct laryngoscopy by an experienced anesthesiologist blinded to group allocation, just before intubation. Investigators responsible for data collection and patient follow-up were also blinded, and patients were unaware of their group assignments. Randomization codes were revealed only after statistical analyses were completed.

Anesthesia was administered according to a standardized protocol [3]. Before intubation, the oropharyngeal cavity and hypopharynx were sprayed with four puffs of the assigned spray. Tracheal intubation was performed using a stylet-guided technique with single-use polyvinyl chloride endotracheal tubes (Bıçakçılar, İstanbul, Türkiye) with low-pressure, high-volume, soft-seal cuffs (7.0–7.5 mm for females; 8.0–9.0 mm for males). Cuff pressure was maintained between 18 and 22 cmH_2_O using a handheld pressure gauge (Endotest; Rüsch, Kernen, Germany). All patients received 500 mg of intravenous acetaminophen before extubation, and four additional puffs of the assigned spray were administered to the oropharynx after the final oropharyngeal aspiration.

The severity of POST was assessed using the Visual Analog Scale (VAS), a 10-mm line anchored by descriptive extremes such as “no pain” and “worst pain imaginable.” Patients marked their perceived pain intensity at 1 h, 6 h, 24 h, and 1 week postoperatively. Patients were further categorized into two subgroups based on surgical duration: less than 120 min (<120 min) and 120 min or more (≥120 min).

The study protocol received approval from the Institutional Review Board (Decision No: 1705).

## 3. Statistical analysis

A sample size calculation using the OpenEpi website^1^ determined that 30 patients per group were required to detect a 50% reduction in the incidence of sore throat with 80% power at a 5% significance level; 35 patients per group were enrolled to account for dropouts. Statistical analysis was performed using SPSS v.22.0 for MacOS (SPSS, Chicago, IL), with significance set at p < 0.05. Normality was assessed using the Kolmogorov–Smirnov test; nominal data were analyzed using Pearson’s χ^2^ test with Bonferroni correction, and differences between treatment groups were examined using one-way ANOVA with Tukey’s post hoc test.

## 4. Results

Of the 105 patients initially enrolled, nine were excluded from the final analysis (four from the placebo group, three from the benzydamine spray group, and two from the flurbiprofen group) due to multiple intubation attempts (n = 3), the need for additional analgesics (n = 2), and postoperative vomiting during follow-up (n = 4). The study flow diagram is shown in Figure.

The mean age of the patients was 35.04 ± 13.0 years (range: 18–65 years). Among the 96 patients included in the final analysis, 52 (54.17%) were male and 44 (45.83%) were female. Regarding surgical duration, 55 patients (57.29%) had procedures lasting less than 120 min, while 41 patients (42.71%) underwent surgeries exceeding 120 min. Patient characteristics are summarized in Table 1.

Postoperative VAS scores at 1 h, 6 h, and 24 h were significantly lower in the benzydamine HCl, chlorhexidine digluconate, and cetylpyridinium chloride oral spray and flurbiprofen oral spray groups compared to the control group. Although most cases of POST tend to resolve within the first 24–48 h, we included assessments up to postoperative 1-week to capture any prolonged or late-onset symptoms that might not be evident in the early period. At the 1-week follow-up, although the VAS score remained lower in the treatment groups, the difference was not statistically significant. The mean sore throat severity scores and p-values according to groups are summarized in Table 2.

When comparing the benzydamine HCl, chlorhexidine digluconate, and cetylpyridinium chloride oral spray group with the flurbiprofen oral spray group, the VAS scores were lower in the benzydamine group at 1 h, 6 h, and 1 week, but these differences were not statistically significant (p = 0.739, p = 0.585, and p = 0.545, respectively). At 24 h, the flurbiprofen group had a lower VAS score than the benzydamine HCl, chlorhexidine digluconate, and cetylpyridinium chloride group (p = 0.766) (Table 2).

Regarding surgical duration, the VAS scores were significantly lower at 1 h and 6 h postoperatively in the group with surgeries lasting less than 120 min compared to those with surgeries exceeding 120 min (p = 0.035, p = 0.032, respectively). Although the VAS scores at 24 h and 1 week were lower in the shorter-duration group, these differences were not statistically significant (p = 0.081, p = 0.419, respectively).

In patients with a surgery duration of less than 120 min, at 1 h, 6 h, and 24 h postoperatively, the VAS scores were highest in the placebo group, followed by the flurbiprofen group, and lowest in the benzydamine HCl, chlorhexidine digluconate, and cetylpyridinium chloride group. However, only the comparisons between benzydamine HCl, chlorhexidine digluconate, and cetylpyridinium chloride and placebo, as well as flurbiprofen and placebo, were statistically significant. The mean sore throat severity scores and p-values according to groups in patients with surgery duration less than 120 min are summarized in Table 3.

In patients with a surgery duration of more than 120 min, at 1 h, 6 h, and 24 h postoperatively, the VAS scores were highest in the placebo group, followed by the benzydamine HCl, chlorhexidine digluconate, and cetylpyridinium chloride group, and lowest in the flurbiprofen group. However, only the following comparisons were statistically significant: at 1 h, placebo vs. benzydamine HCl, chlorhexidine digluconate, and cetylpyridinium chloride (p = 0.001) and placebo vs. flurbiprofen (p < 0.001); at 6 h, placebo vs. benzydamine HCl, chlorhexidine digluconate, and cetylpyridinium chloride (p = 0.027) and placebo vs. flurbiprofen (p = 0.002); at 24 h, placebo vs. flurbiprofen (p = 0.035). The mean sore throat severity scores and p-values according to groups in patients with surgery duration more than 120 min are summarized in Table 4.

Female patients had higher VAS scores than male patients at 1 h, 6 h, 24 h, and 1 week; however, none of these values were statistically significant (p = 0.247, p = 0.296, p = 0.057, p = 0.481, respectively). When comparing based on BMI, patients with a BMI <25 had lower VAS scores at 1 h, 6 h, and 24 h, and 1 week. Nevertheless, none of these differences were statistically significant (p = 0.973, p = 0.301, p = 0.711, p = 0.610, respectively).

## 5. Discussion

POST remains a prevalent and distressing complication following general anesthesia, significantly affecting patient comfort and satisfaction [2]. In this prospective, randomized, placebo-controlled trial, we found that both NSAID-containing sprays—namely benzydamine hydrochloride and flurbiprofen—significantly reduced the severity of POST compared to placebo. However, the addition of antimicrobial agents, including chlorhexidine digluconate and cetylpyridinium chloride, did not provide any additional benefit.

The etiology of POST and postoperative hoarseness primarily involves irritation, inflammation, and trauma caused by endotracheal intubation and cuff inflation. As such, antiinflammatory medications, including nonsteroidal antiinflammatory drugs (NSAIDs) and corticosteroids, have been extensively studied for their ability to mitigate these complications [7]. Our findings confirm the efficacy of NSAID-based throat sprays in reducing POST severity. Both benzydamine hydrochloride and flurbiprofen demonstrated statistically significant reductions in VAS scores compared to placebo in the early postoperative period. These results align with previous studies highlighting the effectiveness of NSAIDs in alleviating pharyngeal inflammation and discomfort through the inhibition of cyclooxygenase-mediated prostaglandin synthesis [21]. The observed reduction in POST with these agents is consistent with prior research demonstrating the analgesic and antiinflammatory effects of NSAID-based throat sprays [7].

In the literature, only a limited number of studies have investigated the addition of antiseptics to NSAID-containing drugs for the treatment of POST [22–24]. Based on the available data, two studies utilized sprays containing benzydamine hydrochloride combined with chlorhexidine gluconate, while one study examined flurbiprofen combined with chlorhexidine gluconate. These studies demonstrated that the use of these sprays significantly reduced the severity of POST compared to the placebo. However, it remains unclear which active ingredient in the spray is responsible for this effect. This ambiguity arises from the fact that the combinations were evaluated as a whole rather than assessing the individual contributions of each component. In this context, our study is the first in the literature to explore the impact of adding antiseptics to NSAID-containing spray formulations. Our findings suggest that the contribution of antiseptics to reducing POST severity may be limited in the early postoperative period. This challenges the necessity of adding antiseptics and highlights the need for careful consideration when formulating such sprays for indications like POST. Furthermore, our results contribute to the development of simpler and more cost-effective treatment approaches by reducing the use of unnecessary components in POST management.

Our findings did not support the hypothesis that antimicrobial agents could further reduce POST by limiting bacterial colonization and its potential contribution to pharyngeal inflammation. The addition of chlorhexidine digluconate and cetylpyridinium chloride to benzydamine hydrochloride did not provide superior pain relief compared to flurbiprofen alone. This suggests that bacterial colonization may not play a significant role in the acute inflammatory process responsible for POST in the immediate postoperative period. Additionally, the potential antiseptic effects of these agents may not be sufficient to counteract the mechanical and inflammatory trauma induced by endotracheal intubation.

Although slight differences in VAS scores were noted between the benzydamine spray and the flurbiprofen spray groups, these differences did not reach statistical significance at any postoperative time point. This finding indicates that both NSAID-based formulations offer comparable efficacy in managing POST, allowing clinicians to base their choice on factors such as patient preference, availability, or contraindications. Moreover, the lack of additional benefit from antimicrobial inclusion raises concerns about the necessity of antiseptic components in these formulations for the treatment of POST, particularly given their potential to disrupt the normal oral flora.

Surgical duration emerged as a significant factor influencing POST severity [4]. In our study, patients who underwent procedures lasting more than 120 min exhibited notably higher VAS scores at 1 h and 6 h postoperatively. This aligns with previous findings linking prolonged intubation to increased mucosal irritation and inflammation [25]. However, the disparity in VAS scores between shorter and longer surgical durations diminished over time, highlighting the early postoperative period as the most critical window for intervention.

This study has several strengths, including its randomized, placebo-controlled design and the use of a standardized anesthesia protocol to minimize confounding factors. Additionally, the blinding of patients, investigators, and anesthesiologists enhances the validity of our findings. However, certain limitations should be acknowledged. First, as the study was conducted in a single institution, its generalizability may be limited. Second, although our sample size was adequately powered to detect significant differences between groups, larger multi-center trials are necessary to further validate these results. Lastly, microbiological assessments were not performed, preventing an evaluation of whether antimicrobial agents influenced bacterial colonization in the oropharyngeal or endotracheal regions.

## 6. Conclusion

In conclusion, our findings indicate that while NSAID-containing oral sprays effectively reduce the severity of postoperative sore throat, the addition of antimicrobial agents does not offer additional analgesic benefit. Since both the benzydamine hydrochloride–chlorhexidine digluconate–cetylpyridinium chloride combination and flurbiprofen alone demonstrated comparable efficacy, clinical decisions may be guided by patient preference and individual patient factors. Further studies are warranted to identify alternative or adjunctive strategies for preventing POST, particularly in patients undergoing longer surgical procedures.

## Figures and Tables

**Figure:**
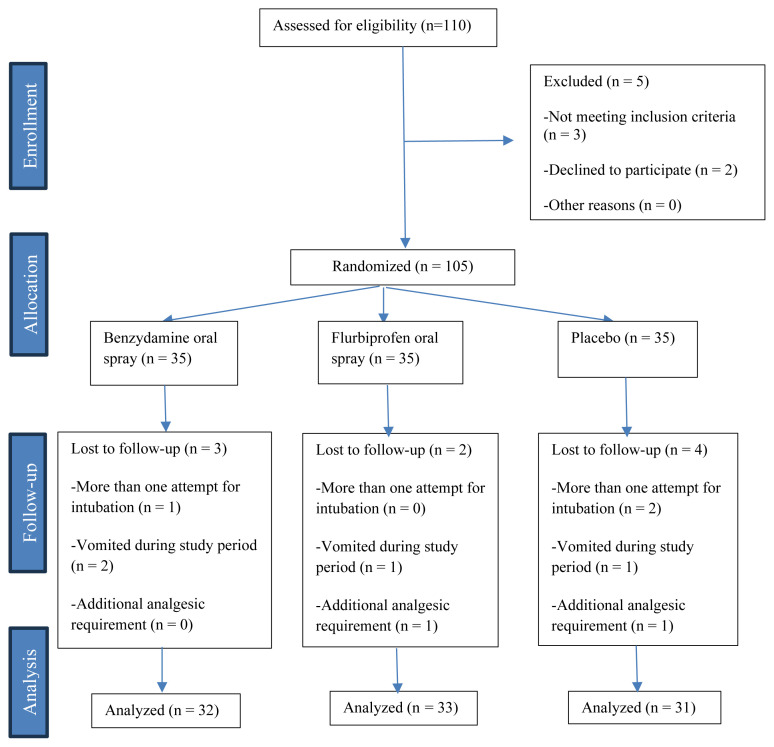
Flow diagram of the study design and patient inclusion process.

**Table 1:** Characteristics of the study population.

Parameters	Group B (n=32)	Group F (n=33)	Group P (n=31)	p-value
**Demographics**				
Sex, M/F	14/18	19/14	19/12	0.335
Age, years	33.03±12.28	34.79±13.33	37.39±17.39	0.414
Weight, kg	71.19±14.28	73.85±17.77	71.16±10.56	0.696
Body Mass Index, kg/m^2^	25.15±4.44	25.96±5.06	24.39±4.01	0.389
**Operative parameters**				
Operation time (<2 h/>2 h)	18/14	21/12	16/15	0.966

Values are given as mean ± SD or number of patients.

* p < 0.005 was accepted for statistical significance (post hoc Tukey’s test).

B = Benzydamine hydrochloride, chlorhexidine digluconate, and cetylpyridinium chloride oral spray, F = Flurbiprofen oral spray, P = Placebo.

**Table 2:** Mean sore throat severity scores of the groups.

				p-value		
Time	Group B (n=32)	Group F (n=33)	Group P (n=31)	Group F vs Group P	Group B vs Group P	Group F vs B
1 h	18.75±18.22	20.91±19.62	50.00±28.01	<0.001	0.001	0.739
6 h	11.56±16.83	13.03±15.25	34.94±30.03	0.005	0.003	0.585
24 h	4.34±9.00	3.36±5.47	16.03±22.04	0.040	0.020	0.766
1 week	0.31±1.76	1.16±5.03	1.61±6.37	0.948	0.514	0.545

Values are given as mean ± SD or number of patients.

* p < 0.005 was accepted for statistical significance (post hoc Tukey’s test).

B = Benzydamine hydrochloride, chlorhexidine digluconate, and cetylpyridinium chloride oral spray, F = Flurbiprofen oral spray, P = Placebo.

**Table 3:** Mean sore throat severity scores for surgeries <2 h.

				p-value		
Time	Group B (n=32)	Group F (n=33)	Placebo (n=31)	Group F vs Group P	Group B vs Group P	Group F vs Group B
1 h	12.78±11.14	21.67±19.57	40.94±31.10	<0.001	<0.001	0.691
6 h	6.00±9.38	13.33±15.51	27.62±32.89	0.006	0.002	0.676
24 h	2.11±6.48	3.38±5.66	14.06±24.37	0.033	0.018	0.794
1 week	0.00	1.29±5.89	1.25±5.00	0.921	0.527	0.588

Values are given as mean ± SD or number of patients.

* p < 0.005 was accepted for statistical significance (post hoc Tukey’s test).

B = Benzydamine hydrochloride, chlorhexidine digluconate, and cetylpyridinium chloride oral spray, F = Flurbiprofen oral spray, P = Placebo.

**Table 4:** Mean sore throat severity scores for surgeries >2 h.

				p-value		
Time	Group B (n=32)	Group F (n=33)	Placebo (n=31)	Group F vs Group P	Group B vs Group P	Group F vs B
1 h	26.43±22.73	19.57±20.50	59.67±21.25	<0.001	0.001	0.702
6 h	18.71±21.51	12.50±15.44	42.73±25.44	0.002	0.027	0.674
24 h	7.21±11.07	3.33±5.36	18.13±19.88	0.035	0.179	0.489
1 week	0.71±2.67	0.91±3.01	2.00±7.74	0.854	0.819	0.994

Values are given as mean ± SD or number of patients.

* p < 0.005 was accepted for statistical significance (post hoc Tukey’s test).

B = Benzydamine hydrochloride, chlorhexidine digluconate, and cetylpyridinium chloride oral spray, F = Flurbiprofen oral spray, P = Placebo.

## Footnotes

1
http://www.openepi.com/SampleSize/SSCC.htm
